# Towards directed evolution of rare earth element binding peptides using genetically encoded sensitizing amino acids

**DOI:** 10.1093/protein/gzag026

**Published:** 2026-09-17

**Authors:** Riley M Bednar, Jason G Marmorstein, Kathleen J Stebe, E James Petersson

**Affiliations:** Department of Chemistry, University of Pennsylvania, 231 S. 34 Street, Philadelphia, PA 19104, United States; Department of Chemistry, University of Pennsylvania, 231 S. 34 Street, Philadelphia, PA 19104, United States; Department of Chemical and Biomolecular Engineering, University of Pennsylvania, 220 South 33rd Street, Philadelphia, PA 19104, United States; Department of Chemistry, University of Pennsylvania, 231 S. 34 Street, Philadelphia, PA 19104, United States; Department of Biochemistry and Biophysics, Perelman School of Medicine, University of Pennsylvania, 421 Curie Boulevard, Philadelphia, PA 19104, United States

**Keywords:** rare earth elements, lanthanide binding peptide, bacterial display, directed evolution, genetic code expansion, luminescent resonant energy transfer

## Abstract

As demand for the rare earth elements (REEs) that power new and emerging technologies increases rapidly, there is a growing need for next-generation separations systems that can provide more selective isolation of these critical, but difficult-to-purify materials. Due to their evolvability and compatibility with existing separations platforms, polypeptides represent an ideal and environmentally-friendly chassis with which to meet this challenge; however, there exist no high-throughput approaches to selecting polypeptides with improved affinity and selectivity towards the various REEs, chiefly due to the difficulty of detecting a selectable phenotype. Here, we present work towards overcoming this obstacle by developing a bacterial display platform for the direct sensing of REE binding in living bacterial cells. Our approach exploits the antenna effect, which allows a sufficiently proximal fluorophore to sensitize a bound europium ion and stimulate emission through resonant energy transfer, and makes use of genetic code expansion to insert a fluorescent amino acid directly into the polypeptide backbone *in cellulo*. We find that targeting a model lanthanide-binding peptide to the periplasm enables the accumulation of sufficient concentrations of this binder in an easily accessible subcellular localization such that robust signal can be detected. Through optimization of encoding, sensitization, and handling, we further improve our signal up to >20-fold over control cells lacking binders and demonstrate facile differentiation and characterization of several variant peptides. Our work paves the way for high-throughput synthetic evolution of REE binding polypeptides with expanded functionality.

## Introduction

Rare Earth Elements (REEs)—the 15 lanthanides in addition to scandium and yttrium—are integral components of numerous emerging technologies, yet new separation paradigms are needed to meet rapidly growing demand. Due to their unique luminescent, magnetic, and electronic properties, REEs are indispensable for applications spanning consumer electronics, energy, transportation, and medicine (Zhao et al., 2025), and are central to the green energy transition (Depraiter & Goutte, 2023). Despite this increasing demand, global supply remains limited, with China producing >90% of REEs, sparking renewed interest in new isolation and recycling technologies, particularly from the U.S. Department of Energy (Humphries, 2013; Energy Department Announces $134 Million in Funding to Strengthen Rare Earth Element Supply Chains, Advancing American Energy Independence, n.d.). Moreover, current REE production relies on intensive and environmentally-destructive leaching and multi-stage solvent extraction using poorly selective extractants to isolate REEs from complex mixtures to the purity required for industrial applications (Royen & Fortkamp, 2016; Gkika et al., 2024). As such, sustainable and highly selective separation technologies are urgently needed.

Polypeptides, with their precisely folded tertiary structures, offer an ideal bio-inspired platform for selective REE purification. As a separation platform, polypeptides provide several advantages, including direct detection of bound REEs *via* energy transfer (ET) through the antenna effect (Sculimbrene & Imperiali, 2006), reversible folding for capture/release (Baldwin, 2007), and compatibility with diverse separation modalities such as resins, hydrogels, and surfactants (Wong et al., 2009). Indeed, REE-binding polypeptides have been described, including the lanthanide binding tag (LBT) developed by the Imperiali group (Sculimbrene & Imperiali, 2006), and the naturally occurring LanM protein identified by the Cotruvo group, which exhibits greater than 10^8^-fold selectively for lanthanides over calcium, despite similar ionic radii (Cotruvo Jr et al., 2018). Although these lanthanide binding polypeptides (LBPs) exhibit high affinity, they have limited selectively among REE species. However, the recent discovery of lanthanide-dependent metalloenzymes with preference for light lanthanides (Daumann, 2019) suggests that either these enzymes or the import machinery (Larrinaga et al., 2024) is capable of distinguishing between REEs, demonstrating that engineering REE-selective LBPs is within grasp.

In this context, a defining advantage of using polypeptides for REE separation is their evolvability. Because they are genetically encoded, LBPs can be diversified through synthetic evolution approaches to tune affinity and specificity (Fig. 1a). Despite this potential, no truly high-throughput platform (≥10^6^ variants) exists for selecting REE-binding polypeptides. An early combinatorial approach by Imperiali and colleagues leveraged tryptophan-mediated terbium sensitization in a synthetic peptide library; however, the absence of a genotype–phenotype linkage required manual bead sorting and sequencing, limiting throughput to several thousand members despite a potential library size of >10^17^ members for a sequence of ~15 amino acids (Nitz et al., 2003). A recent DNA-based diversification in *E. coli* established a genotype–phenotype linkage, but required cell lysis due to cell-incompatible UV-based tryptophan sensitization, restricting screening to 96-well formats (Klassen et al., 2025). Genetic encoding of 2-acridonylalanine (Acd)—a technique pioneered by our laboratory (Marmorstein et al., 2024)—bypasses this limitation by enabling blue-light sensitization. Wang and coworkers used this approach for direct europium detection, but cytosolic expression of LanM required high Eu^3+^ concentrations and a colony-based screen to compensate for low signal, limiting throughput to ~10^5^ variants (Wang et al., 2025). In contrast, bacterial display provides a robust genotype–phenotype linkage enabling the diversification and selection of polypeptides by displaying them in an accessible location on the cell exterior and, when paired with high-throughput selection formats like fluorescent-activated cell sorting (Luo et al., 2023), enables screening of >10^8^ cells (Löfblom, 2011).

**Figure 1:**
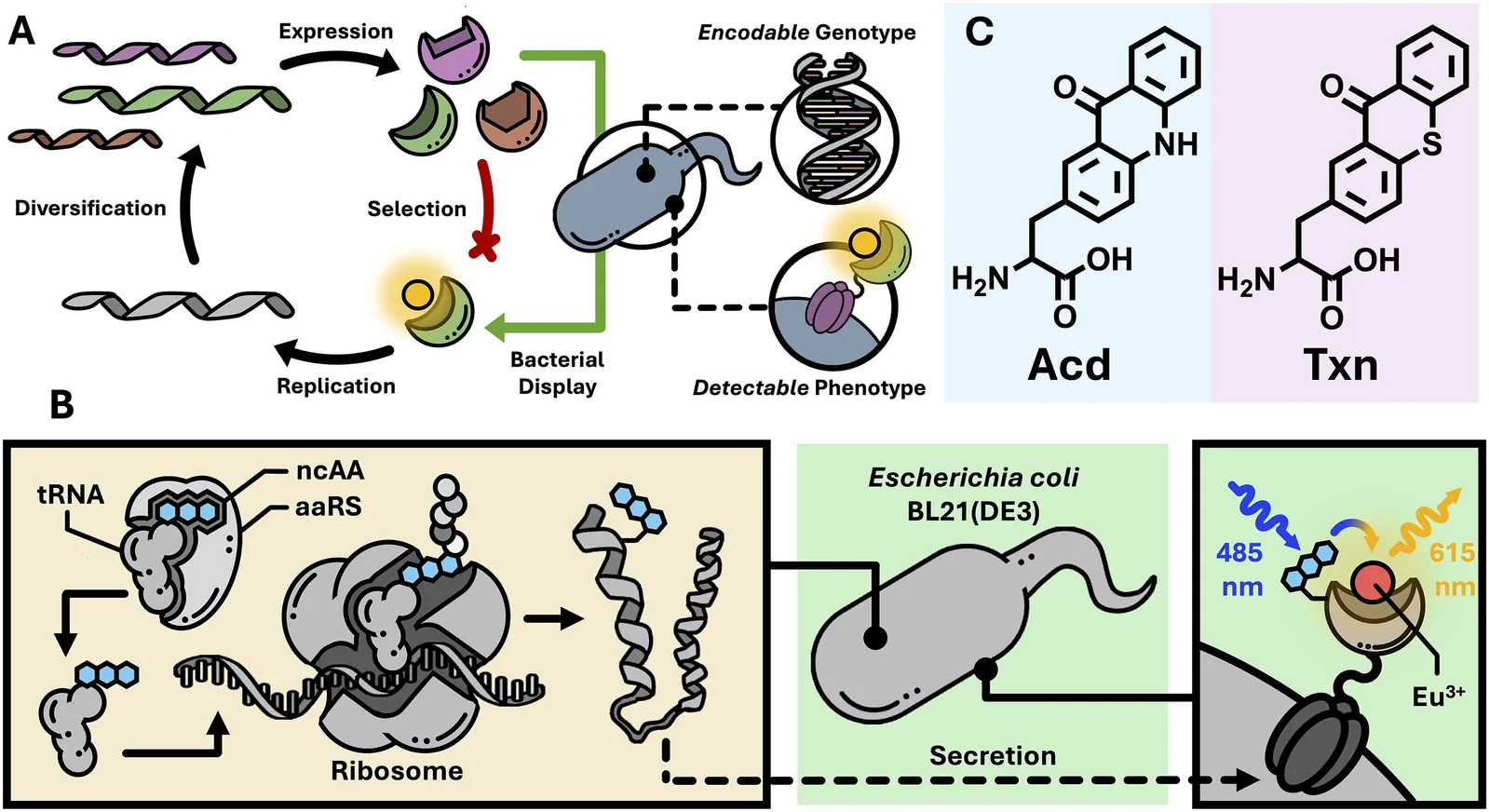
Conceptual design of an *in cellulo* detection platform for REE binding. (A) schematic depicting the critical steps in any directed evolution campaign, specifically bacterial display. Note, an inability to detect REE binding in live cells prevents selection of improved binders, disrupting the cycle; however, as a requisite for directed evolution, the *E. coli* cell provides a robust genotype–phenotype linkage. (B) schematic of the proposed *in cellulo* detection platform, with an emphasis on the features thought to be necessary for the observation of REE binding. (C) structures of the two ncAAs, 2-acridonylalanine (Acd) and 2-thioxanthonylalanine (Txn), used in this study.

Here, we report a robust, truly *in cellulo* REE-sensing platform, the first such system based on periplasmic bacterial display with genetically encoded noncanonical amino acid (ncAA) sensitizers of Eu^3+^ emission. Using genetic code expansion (GCE), we site-specifically incorporated Acd and another sensitizer, 2-Thioxanthonylalanine (Txn) (Fig. 1c) into the antenna position of LBPs to sensitize Eu^3+^ emission *via* ET. Targeting LBP to the periplasm—an accessible compartment that supports high-level accumulation when fused to maltose-binding protein (MalE) (Fig. 1b)—greatly enhanced luminescent signal. We further improved performance through optimizing expression and substitution of Acd with Txn. Using our platform, we show that LBPs of varying affinity can readily be differentiated and binding by non-luminescent REEs can be inferred through competitive displacement. Our findings establish the feasibility and versatility of *in cellulo* REE sensing and lay the groundwork for high-throughput synthetic evolution campaigns across extended sequence space, setting the stage for the discovery of LBPs with expanded functionality.

## Results and discussion

### Periplasmic display of LBPs enables in cellulo REE sensing

To enable detection of bound REEs, we genetically encoded Acd into the model LBT1 (Nitz et al., 2003) peptide (hereafter refer to as LBT1^Acd^) that was fused to various protein carriers, as previously described (Marmorstein et al., 2024). The native tryptophan at position 9 was substituted for a UAG codon (referred to as LBT1^UAG^), which in combination with the commonly used *Methanocaldococcus jannaschii* tyrosyl aminoacyl tRNA synthetase tRNA pair specific for Acd (*Mj*AcdRS/tRNA_CUA_) (Sungwienwong et al., 2017), enables direct incorporation of this ncAA into the LBP during translation. Positioned here in close proximity to bound REEs, Acd functions as a sensitizer, enabling ET through excitation of Acd at ~385 nm and stimulated emission by Eu^3+^ at ~615 nm (Marmorstein et al., 2024) (Figs. 1 and S1). Eu^3+^ was selected as our model REE due to its spectral compatibility with Acd (Ortuno Macias et al., 2024).

Initially, we envisioned a bacterial display system where LBT1 localized to the *E. coli* outer membrane. To achieve this, constructs were generated using three established anchoring proteins: Lpp-OmpA, AIDA-I, and InaK (van Bloois et al., 2011; Nicchi et al., 2021; Mahdavi et al., 2022). However, previous studies that displayed LBPs on the surface of *E. coli* or *Caulobacter crescentus* for bioadsorption used constructs with up to 16 tandem copies of the LBP to achieve appreciable signal (Park et al., 2016; Park et al., 2017), which is incompatible with our directed evolution efforts due to the difficulty in cloning repetitive LBP copies with the same constellation of mutations in each consecutive repeat. Anticipating low signal arising from limited surface area of the outer membrane, we opted to target LBT1 to the periplasm, the region between the inner and outer membranes of gram-negative bacteria (Fig. 2a). We reasoned that the periplasm, which is accessible to hydrophilic molecules below ~650 Da (Krishnamoorthy et al., 2016) including europium and other lanthanides (Bayer & Bayer, 1991), might offer a larger three-dimensional volume (~20% of the total cell volume by some estimates (Graham et al., 1991) than the heavily crowded (Sun et al., 2022) two-dimensional outer membrane, thereby improving signal. Therefore, LBT1^UAG^ was inserted with a Gly-Ser linker to the N-terminus of maltose binding protein (MalE)—the most abundant periplasmic protein in *E. coli* (Han et al., 2014)—which should yield scarless LBT1^Acd^-MalE after processing (Zhang et al., 2025). As a control, we also generated a LBT1^UAG^-MalE construct lacking the signal peptide that should localize to the cytoplasm.

**Figure 2:**
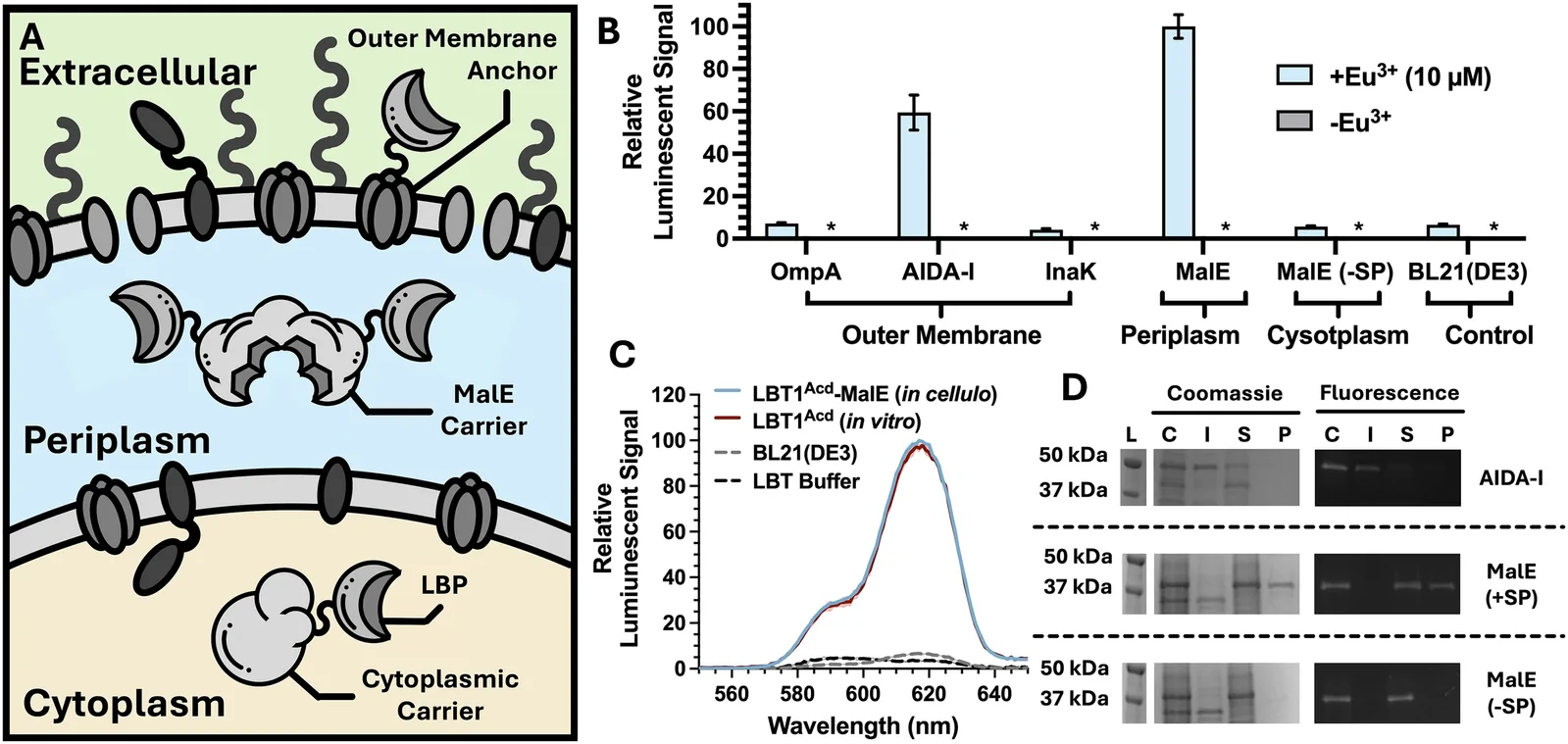
Comparisons of LBP localization. (A) schematic of various subcellular localizations to which the model LBT1 peptide was targeted through fusion to various carrier proteins. (B) comparison of luminescent signal for LBT1 targeted to various subcellular localizations in the presence (light blue bars) or absence (gray bars; ^*^indicates that signal is close to baseline) of 10 μM Eu^3+^. Relative luminescent signal is presented as the luminescent signal at 615 nm normalized to the highest observed signal. (C) *in cellulo* emission spectra of Eu in the presence of *E. coli* BL21(DE3) either expressing (solid blue line) or not expressing (dashed gray line) LBT1^Acd^-MalE in the presence of 10 μM Eu^3+^. Purified LBT1^Acd^ (solid red line) and LBT buffer (dashed black line) are shown for reference. (D) SDS-PAGE in-gel fluorescence of BL21(DE3) cells expressing either LBT1^Acd^-AIDA-I (52 kDa), or LBT1^Acd^-MalE with (‘+SP’) or without (‘-SP’) signal peptide (46 kDa) arising from various cellular fractionations. The contents of each well are indicated above. Fractionations include crude lysate (‘C’), insoluble fraction (‘I’), soluble fraction (‘S’) and periplasmic fraction (‘P’); ‘L’ indicates protein ladder. For an uncropped image of gels, see Fig. S6. Error bars/envelopes represent the standard deviation of 3 replicates.

To compare these constructs, each was expressed in BL21(DE3) cells grown in 2xYT at 18°C and pre-washed in MES buffer, a standard acidic buffer used for LBT binding studies (Marmorstein et al., 2024), prior to incubation with Eu^3+^. The periplasmic LBT1^Acd^-MalE construct produced the strongest luminescent signal (~15-fold above control cells, Fig. 2b) and exhibited an emission spectrum identical to that of purified LBT1^Acd^ (Fig. 2c). Of the outer membrane-targeted constructs, only AIDA-I produced detectable signal (~60% of LBT1^Acd^-MalE; Fig. 2b), while the other two showed negligible signal (likely due to low expression; Fig. S6). Unsurprisingly, the cytoplasmically-localized LBT1^Acd^-MalE showed minimal signal (Fig. 2b); however, lysis did greatly improve signal, consistent with limited Eu^3+^ transport across the inner membrane (Fig. S2). Critically, we also observed that lysis did not markedly increase ET for LBT1^Acd^-MalE in the periplasm, corroborating the outer membrane’s permeability to Eu^3+^ (Fig. S2). We also confirmed that all constructs delivered LBT1 their intended localization (Fig. 2d). Beyond serving as an optimal carrier for *in cellulo* ET sensing, MalE offers additional benefits, including solubility-enhancing and folding-chaperone properties and serves as a purification handle that sidesteps the complicating metal-chelating behavior of other tags and should therefore be useful for downstream applications (Riggs, 2001). Taken together, these results demonstrate that the periplasm is a superior subcellular location for *in cellulo* ET sensing.

### Next-generation genetic code expansion tools further improve luminescent signal

Having demonstrated that facile REE binding detection using our periplasmically-targeted LBT1^Acd^-MalE construct is feasible *in cellulo*, we next wondered if we could further improve detection by taking advantage of several recent GCE innovations. Specifically, the Mehl lab has developed a 3^rd^ generation Acd-specific *Ma*PylRS/tRNA_CUA_ pair (Avila-Crump et al., 2022) derived from *Methanomethylophilus alvus* with improved solubility and stability (Gottfried-Lee et al., 2022; Seki et al., 2020; Beránek et al., 2019) (henceforth referred to as the *Ma*AcdRS/tRNA_CUA_ pair), as well as a new series of plasmids for housing GCE machinery—the pAJE series—that have been engineered to improve encoding efficiency by boosting plasmid copy number (Eddins et al., 2023). Moreover, the Green lab described a thioxanthone-containing ncAA, referred to as Txn, similar in structure to Acd (Fig. 1b), but with a longer-lived triplet state that can provide stronger sensitization (Chen et al., 2025; Crawshaw et al., 2025; Wang et al., 2025). Given the structural similarity of Acd and Txn, we wondered if this next generation *Ma*AcdRS/tRNA_CUA_ pair housed on an optimized pAJE plasmid is permissive to this new sensitizer ncAA.

To test this, we paired both our current *Mj*AcdRS pair (housing variant A9 on a pDule2 plasmid) and a 30 copy-number pAJE plasmid containing the new *Ma*AcdRS A7 variant (referred to as pDule2-*Mj*Acd (A9) and pAJE[30]-*Ma*Acd (A7), respectively) with a SUMO-sfGFP^UAG^ reporter (Eddins et al., 2023) and observed the production of fluorescence as a function of Acd or Txn concentration. In this context, successful encoding enables production of full-length SUMO-sfGFP^ncAA^, resulting in a fluorescence increase that can be normalized relative to SUMO-sfGFP^WT^ and reported as suppression efficiency (Alexander et al., 2025). As can be seen in Fig. 3a, the pAJE[30]-*Ma*Acd (A7) system produced notably more fluorescence than pDule2-*Mj*Acd (A9) at higher ncAA concentrations, while both surprisingly appeared to prefer Txn as a substrate—peaking at around 80% of SUMO-sfGFP^WT^ at 100 μM for *Ma*Acd, 5-fold lower than Acd—despite this not being the substrate for which either aaRS was selected for.

**Figure 3:**
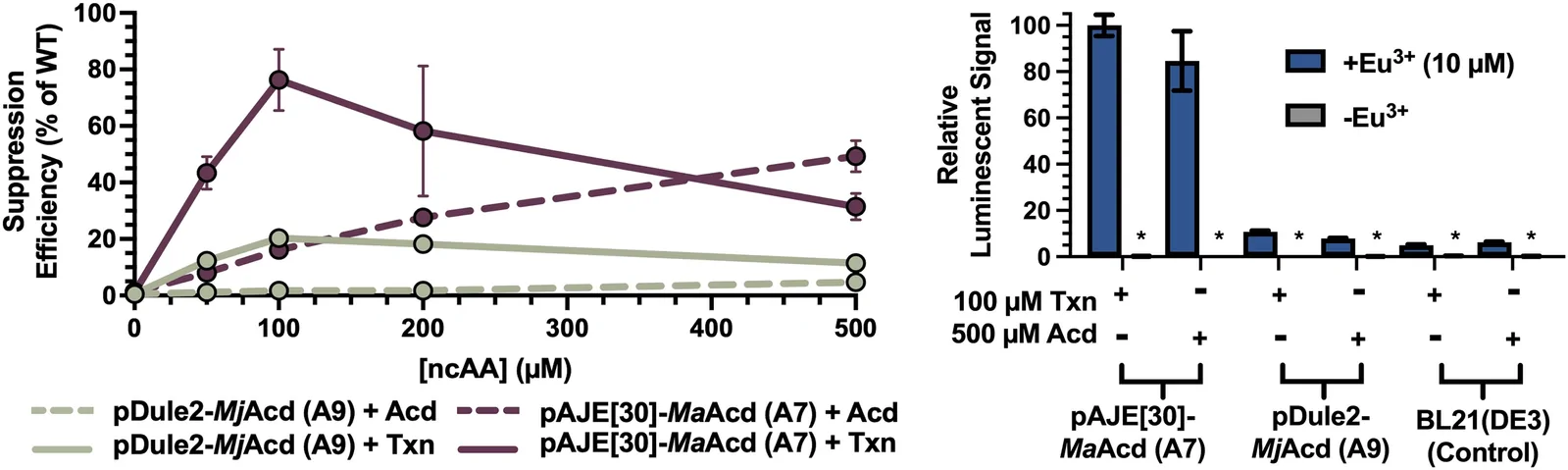
Optimization of GCE systems. (A) assessment of suppression efficiency for two Acd/Txn encoding systems (the 1st-generation *Mj*Acd A9 variant aaRS/tRNA pair housed on a pDule2 plasmid, shown as gray-green lines; and the 2nd-generation *Ma*Acd-based A7 variant aaRS/tRNA pair housed on a pAJE[30] plasmid, shown as maroon lines) at various concentrations of Acd (dashed lines) or Txn (solid lines) using SUMO-sfGFP as a reporter. Suppression efficiency represents the amount of normalized fluorescence produced by either encoding system at a given ncAA concentration relative to the amount of normalized fluorescence produced by a SUMO-sfGFP^WT^ reference control. Normalized fluorescence represents the amount of fluorescence relative to the OD_600_ of the cell suspension. (B) relative luminescent signal of BL21(DE3) housing either pAJE[30]-*Ma*Acd (A7), pDule2-*Mj*Acd (A9), or neither machinery plasmid expressing LBT1^UAG^-MalE in the presence of either 100 μM Txn or 500 μM Acd in AIM at 37°C after exposure to 10 μM Eu^3+^ (blue bars) or absence of REE (gray bars; ^*^indicates that luminescent signal is close to baseline). Error bars represent the standard deviation from 3 replicates.

Given that the screen described above was performed straightforwardly in autoinduction media (AIM) (Studier, 2005) at 37°C without manual induction or thermal shifts, we wondered if optimizing the growth medium could improve and simplify our workflow. We were particularly interested performing direct *in situ* measurements on cells suspended in growth medium without pre-washing, which could simplify future screening efforts by removing a time-consuming and laborious step, especially if combined with autoinduction. Accordingly, we evaluated *in cellulo* ET across three media (2xYT, a rich medium; M9 (Miller, 1972) and AIM, both defined media) at either 18 or 37°C, and with or without pre-washing cells before measurement. However, we observed that Txn, unlike Acd, strongly sensitizes Eu^3+^ in the absence of LBT1^ncAA^ (Fig. S3a), so we performed this comparison using Acd instead. We found that AIM at 37°C yielded the strongest luminescent signal (~20% higher than 2xYT at 18°C), whereas M9 produced negligible signal (Fig. S3b). Unfortunately, *in situ* measurements exhibited minimal ET in AIM and M9 and a high background in 2xYT (Fig. S3b). Since both M9 and AIM are neutral and abundant in phosphate—conditions that can lead to REE precipitation (Klassen et al., 2025; Jordan et al., 2024)—we exposed LBT1^Acd^-MalE-expressing cells to MES buffer prepared at pH 7 and/or with 50 mM PO_4_ and observed that only in the presence of PO_4_ was ET abolished (Fig. S3c). Moreover, we observed that Eu^3+^ experienced a ~ 20-fold increase in ET when added to 2xYT (Fig. S3d), likely due to endogenous sensitizers, underscoring the incompatibility of these media for *in situ* measurements.

Given the clear advantages of AIM, we used this medium to compare our two ncAAs and encoding systems for *in cellulo* Eu^3+^ sensing by pairing each system with LBT1^UAG^-MalE and Txn or Acd at 100 or 500 μM (the optimal concentration for each as per Fig. 3a), respectively. We observed that the pAJE[30]-*Ma*Acd system largely recapitulated the trend from our SUMO^UAG^-sfGFP screen, with ~20% greater signal from Txn relative to Acd and 20-fold above controls cells despite the ~5-fold lower concentration (Fig. 3b). It is unclear why the *Mj*Acd system performs so poorly in AIM, given our prior success in 2xYT (Fig. 3b). Moreover, differences in encoding efficiency prevent a direct confirmation of whether the improvements from Txn encoding *via Ma*Acd result from enhanced expression or Eu^3+^ sensitization. Nevertheless, we decided to proceed with encoding Txn using the pAJE[30-]-*Ma*Acd system while concluding that the choice of encoding system, ncAA, and media have profound effects on *in cellulo* ET detection.

### Optimized encoding systems enable in cellulo characterization of LBPs

A key requirement of any selection platform is that variants with desirable phenotypes be discernible from those with undesirable phenotypes. Therefore, we evaluated whether our *in cellulo* ET system can distinguish LBPs of differing affinities. Five additional LBT variants were generated by scrambling chelating residues (Asp, Glu, Asn) to vary charge and size then evaluated them *in cellulo*. At 10 μM Eu^3+^ all variants were readily distinguishable (Fig. S4a) and possessed distinct dose-dependent responses and plateaus to Eu^3+^ (Fig. 4a), highlighting notable differences in affinity and/or sensitization. Interestingly, we found that these curves were best fit by a sigmoidal model, owing to the absence of response at low Eu^3+^ concentrations which may be due to competitive binding to low-affinity, high-abundance charged moieties present in the cellular environment (Breuker et al., 2020). Curious how *in cellulo* binding compares to *in vitro*, we leveraged the MalE domain to purify all variants using amylose columns and tested their binding *in vitro*, finding that these variants mostly recapitulated *in cellulo* trends (Fig. 4b) and possessed similar spectra (Fig. S4b). Importantly, we noticed that *in vitro* binding curves fit a hyperbolic model with binding at lower Eu^3+^ concentrations (Fig. 4c), supporting our observation that cellular constituents likely nonspecifically bind to Eu^3+^. Interestingly, the D1E variant, while a moderate binder *in cellulo*, does not appear to be so *in vitro*, showing that binding and/or sensitization can be context-dependent.

**Figure 4:**
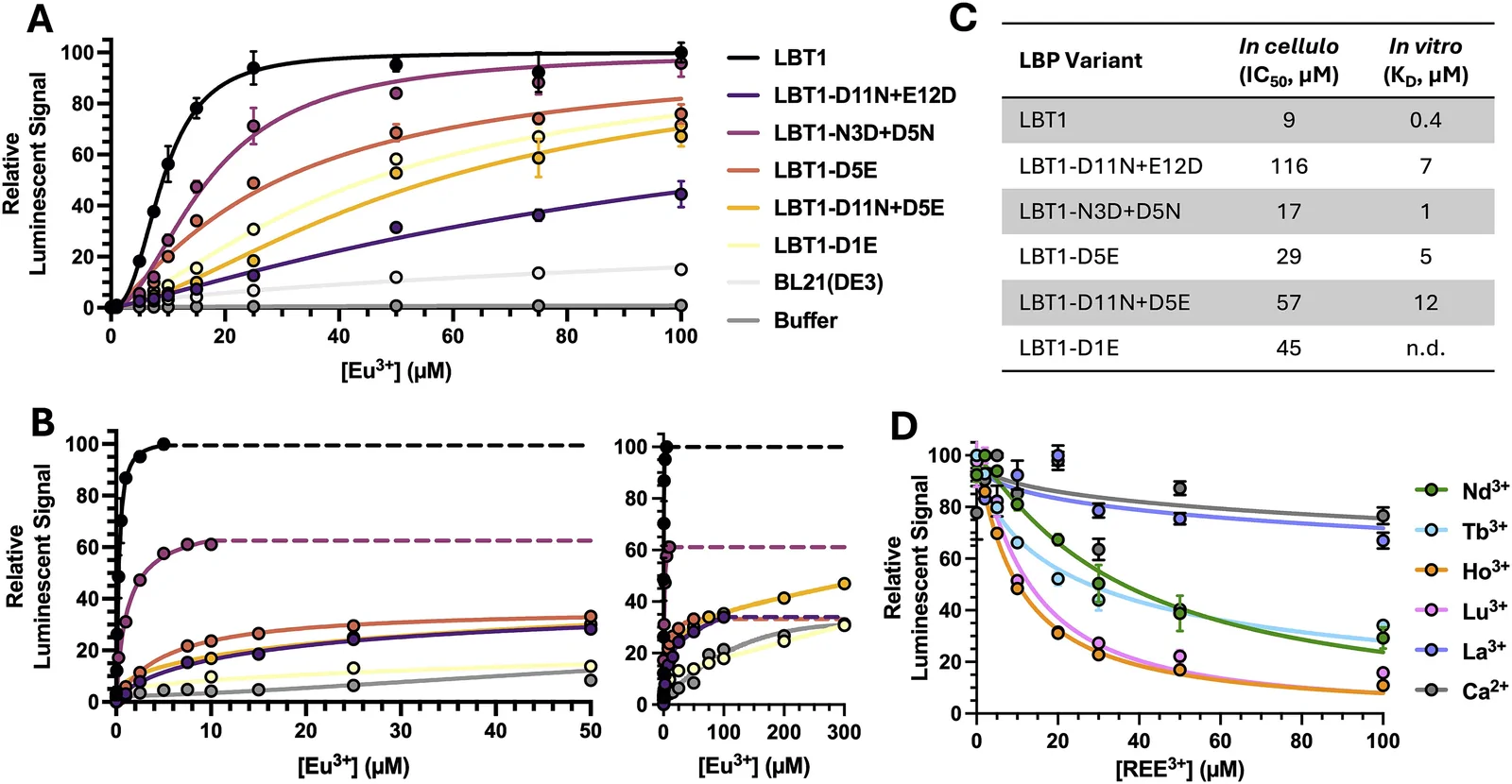
*in cellulo* characterization of various LBP variants. (A) *in cellulo* binding curve of several LBP variants fused to MalE and containing Txn at the antenna position under optimal conditions. All cell suspensions were diluted to an OD_600_ of ~1. See figure legend for curve identities. BL21(DE3) cells not expressing any LBP^Txn^-MalE variant and blank buffer controls are included as solid light gray and dark gray lines, respectively. (B) *in vitro* binding curve of the LBP variants shown in panel (A). all LBPTxn-MalE variants were purified and assayed at 50 nM in LBT buffer. Because the concentration range varied so widely, two regimes (low concentration, left inset; high concentration, right inset) are shown. Curve colors are the same as in a without the BL21(DE3) control, and with dashed lines representing extrapolations beyond the curve fitting range to aid in data interpretation. (C) table summarizing the affinity values observed for each variant in panels (A) (*in cellulo* fit; IC_50_) and (B) (*in vitro* fit; *K*_D_), respectively. N.d. indicates that a regression line could not be fit due to poor signal. (D) *in cellulo* competition assay of LBT1^Txn^-MalE between Eu^3+^ and either Nd^3+^ (green line), Tb^3+^ (blue line), Ho^3+^ (orange line), Lu^3+^ (pink line), La^3+^ (dark blue line), and Ca^2+^ (gray line). Eu^3+^ was held constant at 10 μM. Error bars represent the standard deviation from three replicates. All fit curves have an R^2^ value exceeding 0.98 for all LBT1^Txn^-MalE-containing series.

Having demonstrated that we could characterize Eu^3+^ binding *in cellulo*, we next wondered if we could use our bacterial display platform to infer binding by other REEs. Prior work indicates that binding of REEs not directly sensitized by Acd/Txn can be inferred through signal attenuation via competitive displacement of Eu^3+^  Marmorstein et al., 2024. Accordingly, LBT1^Txn^-MalE-expressing cells were incubated with a fixed concentration of Eu^3+^ and varying concentrations of Nd^3+^, Tb^3+^, Ho^3+^, Lu^3+^, as well as La^3+^ and Ca^2+^. Each ion competed to varying degrees, with Ho^3+^ and Lu^3+^ binding more strongly than Nd^3+^ and Tb^3+^, and La^3+^ and Ca^2+^ producing negligible decreases (Fig. 4d). These trends were also mostly mirrored *in vitro*, with characteristically lower *K*_i_ values when measured *in vitro*, as like above (Fig. S4a-b), similar to previous observations (Ortuno Macias et al., 2024). Interestingly, we observed that Tb^3+^ is slightly sensitized by Txn (Chen et al., 2025) (Figs. S5c-S5f), which may explain its shallower curve in Fig. 4d. Taken together, these results demonstrate that detailed characterizations can be carried out quite robustly *in cellulo* and highlight the sensitivity and versatility of our periplasmic display platform.

## Conclusions

To meet the increasing demand for REEs, bio-inspired polypeptide-based systems that can deliver REE-selective isolation are sorely needed. Here, we address a key limitation in LBP directed evolution—the inability to directly observe REE binding *in cellulo*—through genetically encoding a sensitizer and targeting the LBP to the periplasm. Through optimization of the genetic encoding of a thioxanthone-containing ncAA, we could push luminescent signal to ~20-fold above control cells. Our periplasmic display system enables facile discrimination between LBPs of varying affinities and indirect measuring of other REEs, largely recapitulating *in vitro* trends, but with measured affinities greater *in vitro* than *in cellulo* due to unique features of the cellular environment. These advances overcome a central barrier to large-scale synthetic evolution of LBPs *via* bacterial display. In the future, we plan to employ this platform in support of such endeavors with the hopes of discovering novel LBPs for selective REE enrichment and to gain insight into the principles that drive such specificity.

## Supplementary Material

Bednar_PEDS_Supplemental_Revised_gzag026

## Data Availability

Primary data have been made available in the supplementary file Bednar_PEDS_Data.xlsx.
